# Storage media and not extraction method has the biggest impact on recovery of bacteria from the oral microbiome

**DOI:** 10.1038/s41598-019-51448-7

**Published:** 2019-10-18

**Authors:** Xiaoyan Zhou, Shanika Nanayakkara, Jin-Long Gao, Ky-Anh Nguyen, Christina J. Adler

**Affiliations:** 1 School of Dentistry, Faculty of Medicine and Health, The University of Sydney, Sydney, NSW 2006 Australia; 2 Institute of Dental Research, Westmead Centre for Oral Health, Westmead, NSW 2145 Australia; 3 Charles Perkins Centre, The University of Sydney, Sydney, NSW 2006 Australia

**Keywords:** Microbiome, Bacterial genetics

## Abstract

Next Generation sequencing has greatly progressed the exploration of the oral microbiome’s role in dental diseases, however, there has been little focus on the effect of sample storage conditions and their interaction with DNA extraction method. Dental plaque samples collected from 20 healthy participants were pooled and stored in either 75% ethanol or Bead solution for up to 6-months at −80 °C, prior to DNA extraction with either QIAamp (non-bead beating) or PowerSoil (bead-beating) kit, followed by Illumina sequencing of *16S rRNA gene*. We found that storage media and not extraction method had the biggest influence on the diversity and abundance of the oral microbiota recovered. Samples stored in Bead solution, independent of the extraction kit, retrieved higher diversity (PowerSoil p = 1.64E-07, QIAamp p = 0.0085) and had dissimilar overall ecologies as indicated by lower level of shared diversity (PowerSoil p = 0.0000237, QIAamp p = 0.0088). Comparatively, samples stored in Bead solution and extracted with PowerSoil recovered a higher abundance of *Streptococcus* species. These data indicate that Bead solution can preserve the oral microbiome in dental plaque reliably, for periods of up to 6-months at −80 °C, and is compatible, with either a bead-beating or non-bead beating DNA extraction method.

## Introduction

Consisting of over 700 prevalent taxa at the species level, the oral microbiota is recognised as the second most complex and diverse microbial community in the human body^1,2^ and has been widely studied to unravel its important role in oral^3^ and general health and diseases. The oral microbiota has a symbiotic relationship with the host and disturbing homeostasis of the oral microbial ecosystem is associated with oral diseases such as dental caries^4^, periodontal disease^5,6^ and a variety of systematic diseases including Alzheimer’s disease^7^, diabetes^8^, adverse pregnancy outcome and gastrointestinal disorder^9^.

The oral microbiota colonises a variety of habitats (e.g., teeth, tongue, cheek and saliva), in which the composition of microbiota varies significantly due to differences in key environmental conditions^10^. Of these habitats, dental plaque has been commonly used as a proxy in human oral microbiome studies because it is the precursor and cause of the two most common dental diseases, dental caries^10^ and periodontal diseases^11^. In addition, dental plaque shows features of the classic biofilm and its sample collection is convenient, non-invasive and inexpensive.

With the application of high-throughput, next-generation sequencing (NGS) technologies, our understanding of the human microbiome including the oral microbiota, has been greatly improved. NGS has enabled a relatively unbiased view of the overall composition and function of the oral microbiome to be obtained, which is more accurate than traditional culture-based methods. While there are benefits of using NGS to assess the oral microbiome, experimental bias can be introduced during critical experimental steps. These key steps include: selection of sample storage media, temperature and duration, in addition to the DNA extraction method and region of the 16S rRNA gene amplified (if amplicon-based analysis), all of which may influence the results obtained.

Information about the impact of the overall experimental bias on human dental plaque is crucial for large-scale human population and biobank projects. In these situations, sample integrity becomes an issue, as it is not logistically feasible for researchers to collect and process samples on the same day. Instead, participants are usually recruited at various timepoints and samples are collected and stored for future analysis. In the case of a biobanking project, samples are usually collected and banked until requested by researchers for their study. Thus, a good method for sample storage, including both storage media and length of time of storage, and a subsequent compatible DNA extraction protocol is essential for downstream NGS to accurately recover the oral microbiome.

While there are multiple studies on faeces to evaluate the effect of storage conditions and DNA extraction methods on the gut microbiome^12–16^, there is a limited number of studies examining the impact of these issues on dental plaque^17–20^. As the structure and composition of dental plaque is distinct from gut samples, it is essential to test the impact of key experimental steps on dental plaque directly as opposed to using gut samples as a proxy. For example, a study examining the impact of storage of dental plaque in RNAprotect^18^ found it negatively influenced the diversity of bacteria recovered, whereas assessment of storage of gut samples in a similar product, RNAlater, did not find it to significantly influence the diversity of the gut microbiome^12^.

Current published studies examining the impact of experimental steps on oral microbiome samples intended for NGS have focused on using mock communities, the DNA extraction method used and the 16S region targeted. Recently, Abusleme *et al*. tested the influence of DNA extraction on oral microbial profiles in a mock community and concluded that a method including bead beating was superior to other methods for detecting all seven species in the mock community^17^. Teng *et al*. also reported similar results from their prepared mock community in terms of DNA extraction method, meanwhile they found that 16S primers with regions targeting V3-V4 and V4-V5 seemed to yield more reproducible results than V1–V3^19^. Interestingly, Vesty *et al*. systematically evaluated four widely used microbial DNA extraction methods (PowerSoil DNA Isolation kit, QIAamp DNA Mini kit, Zymo Bacterial/Fungal DNA Mini Prep and phenol:chloroform-based DNA isolation) on human dental plaque as opposed to a mock community, and found bacterial genera in dental plaque did not significantly differ across the DNA extraction methods^20^. These studies highlight the difference in results obtained when assessing the simpler mock communities to the more complex human samples. Yet there are no studies on human dental plaque, assessing the influence of experimental steps over the life of a sample, from collection to storage in media, the length of time in storage and method for DNA extraction.

**Figure 1:**
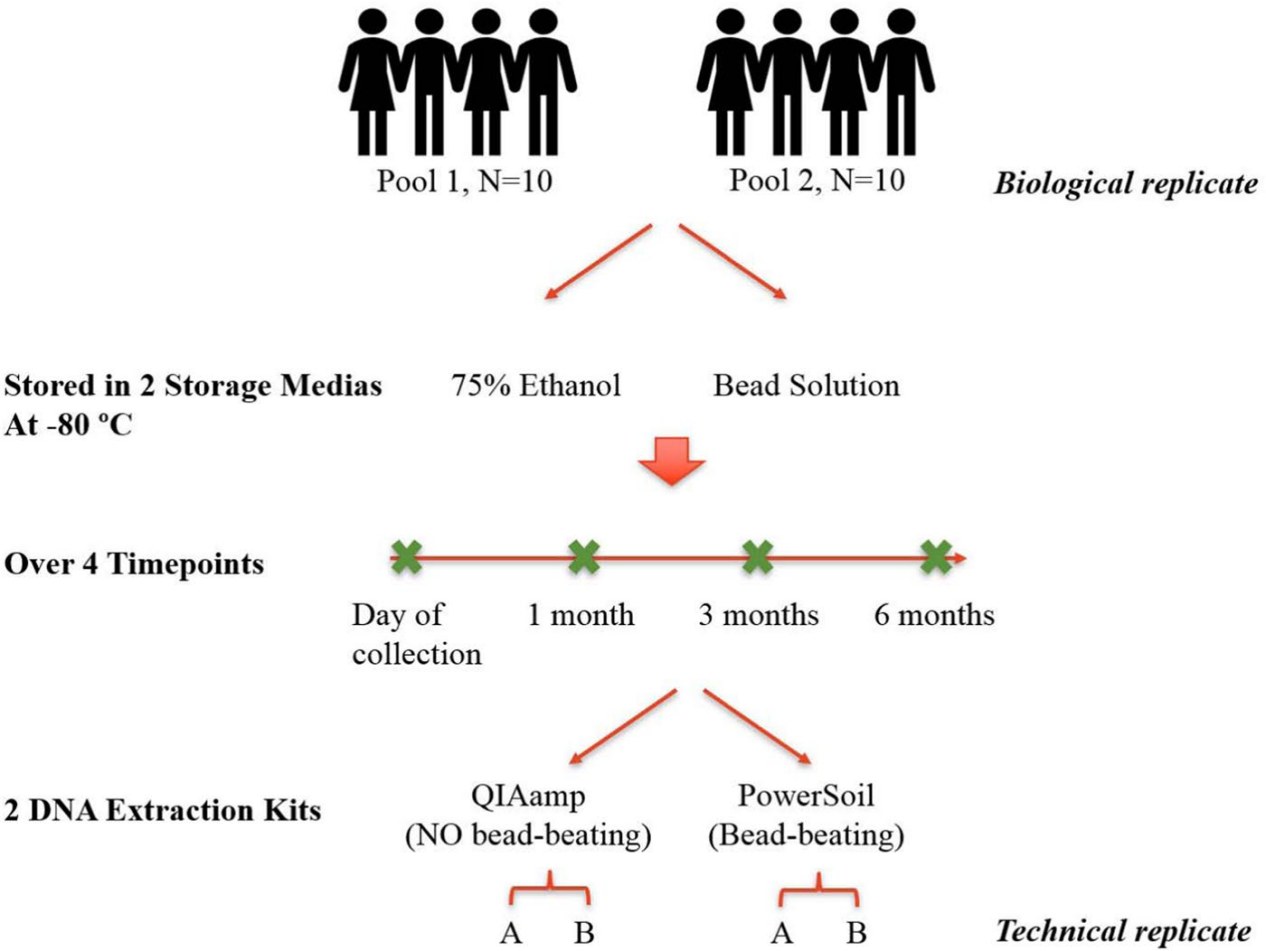
Diagram of experimental design. Dental plaque was collected from 20 healthy human participants, pooled, homogenized and stored in either of two media (75% ethanol or Bead solution) at −80 °C. At each time point (0, 1 month, 3 months and 6 months), samples were subjected to DNA extraction via two commonly used DNA extraction kits (QIAamp and PowerSoil) followed by Illumina sequencing of 16S gene. For all tested conditions, two pools of dental plaque samples were collected as biological replicate and one technical replicate was conducted in the second pool.

Herein, we compared the impact of storage media, length of storage time at −80 °C (being the recommended storage temperature for microbiome material^21^) and DNA extraction method on the recovery of oral microbiota from human dental plaque samples, to ensure findings were applicable to real-world, human studies. We compared two storage media (75% ethanol and Bead solution) and two of the most commonly used DNA extraction kits (PowerSoil and QIAamp), one with and one without a mechanical disruption step, on dental plaque samples either processed immediately or stored at −80 °C for up to 6 months. Overall this equated to testing four conditions (PowerSoil/Bead solution [PB], PowerSoil/75% ethanol [PE], QIAamp/Bead solution [QB], QIAamp/75% ethanol [QE]), with samples assessed at 4 time points over 6 months. To capture both biological and technical variability, 20 individuals were sampled, and pooled into two groups (10 individuals per group), to produce enough plaque for all comparisons, with duplication of the second group’s conditions performed (Fig. 1). To assess the microbial profile from the 48 DNA extracts, we used Illumina sequencing of the V4 region of 16S rRNA gene, in order to evaluate the effect of varying storage conditions and extraction methods on the microbial diversity recovered from human dental plaque.

## Results

**Figure 2:**
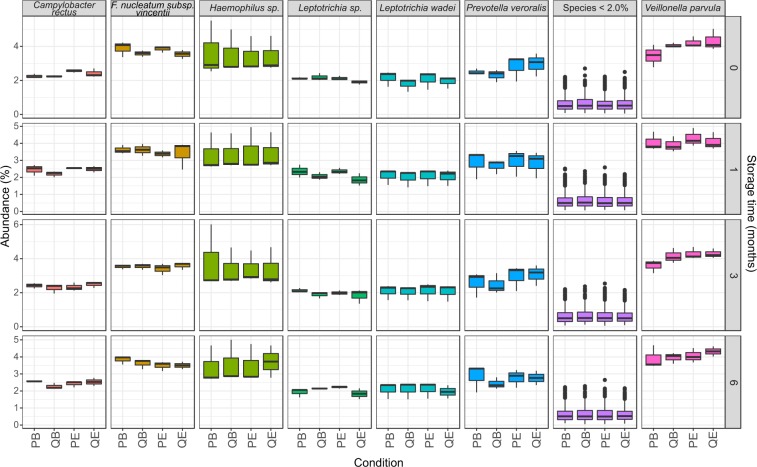
Species abundance grouped by storage media, duration and extraction method. Species with a relative abundance above 2% are displayed, with the remainder of species grouped together (Species <2%) as boxplots, which show the mean and 95% confidence intervals for the data. The data have been normalised for sequence number via cumulative sum scaling (CSS). Abbreviations: PowerSoil/Bead solution (PB), PowerSoil/75% ethanol (PE), QIAamp/Bead solution (QB) and QIAamp/75% ethanol (QE).

Illumina sequencing of the dental plaque extracts (*n* = 48) produced a total of 5,460,928 sequences, with an average of 116,062 sequences per sample, post-quality filtering (Supplementary Fig. 1). One sample in Group T6QE (stored in ethanol and extracted with the QIAamp kit after 6 months storage at −80 °C) was removed from analysis due to low sequence number (6013). The sequences were classified into nine bacterial phyla (Supplementary Fig. 2). For all the storage and extraction conditions, the overall composition of phyla and their abundance was similar. Briefly, the dominant phylum was *Bacteroidetes* (28.7 +/− 1.0%), followed by *Firmicutes* (23.1 +/−0.9%) and *Fusobacteria* (16.9 +/−0.9%). Overall, we found a total of 280 bacterial species or Amplicon Sequence Variants (ASVs), with seven dominant species occurring above 2.0% (Fig. 2, Supplementary Table 1). Of these, the three most prevalent species were *Veillonella parvula* (4.1 +/−0.5%), *Fusobacterium nucleatum subsp. vincentii* (3.6 +/−0.3%) and *Haemophilus sp*. (3.5 +/−1.0%).

### Impact of storage media and DNA extraction method on overall diversity

**Figure 3:**
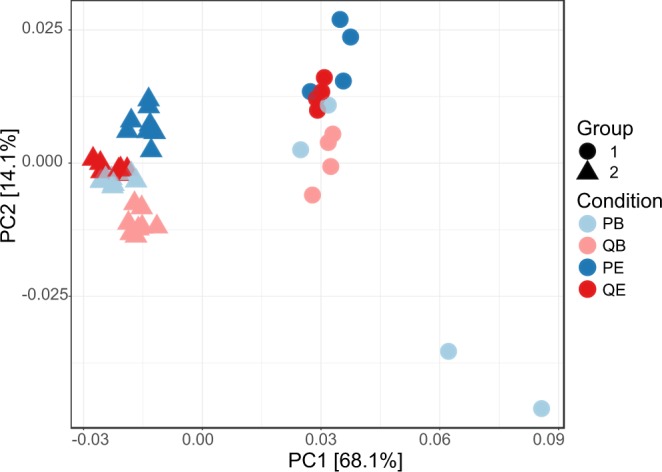
Beta diversity grouped by storage media and extraction method. Beta diversity was calculated from rarefied, ASV representative sequences, using weighted unifrac distances and displayed using Principal Co-ordinates Analysis. Samples are coloured by Condition (storage media and extraction method) and the shape represents the pooled sample group. Abbreviations: PowerSoil/Bead solution (PB), PowerSoil/75% ethanol (PE), QIAamp/Bead solution (QB), QIAamp/75% ethanol (QE), Principal Component 1 (PC1) and Principal Component 2 (PC2).

To assess the influence of the four conditions (PowerSoil/Bead solution [PB], PowerSoil/75% ethanol [PE], QIAamp/Bead solution [QB], QIAamp/75% ethanol [QE]) on overall diversity, we first used an exploratory approach. We compared the shared phylogenetic or beta diversity between all extracts and used these distances to perform a Principal Co-ordinates Analysis (PCoA). The PCoA (Fig. 3) revealed a strong clustering of extracts by sample group along the first principal co-ordinate, representing biological variation between the two pools of samples. The second principal co-ordinate revealed a clustering of samples by condition (storage media and DNA extraction method).

**Figure 4:**
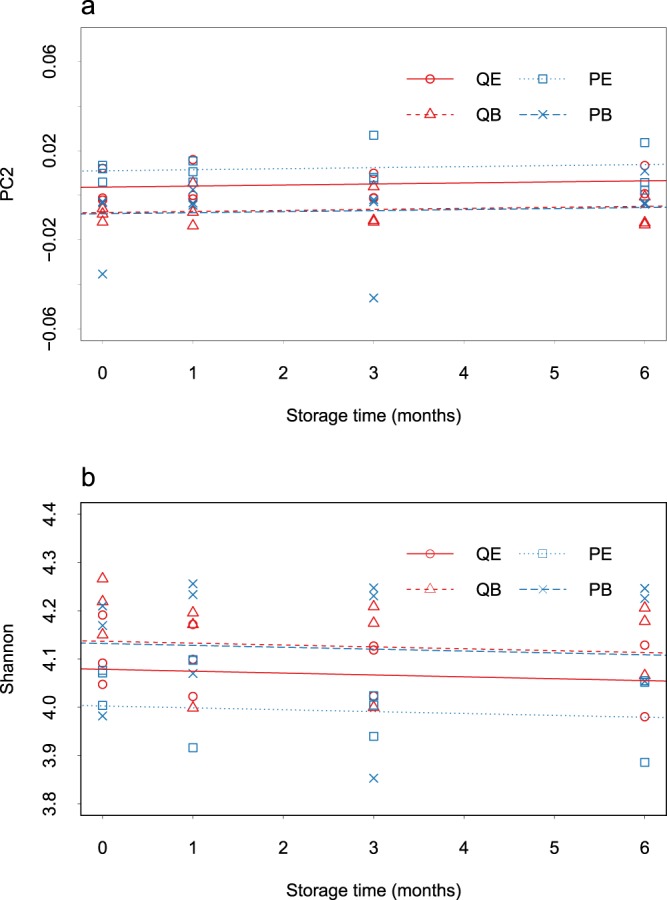
Beta (**a**) and Alpha (**a**) diversity by storage media, duration and extraction method. (**a**) PC2 values displayed in Fig. 3 plotted by storage time. (**b**) Alpha diversity metric, the Shannon Index were calculated in Phyloseq (version 1.20.0) and plotted by storage time displayed. For both (**a**,**b**) the samples are coloured by extraction kit (blue = QIAamp, red = PowerSoil), with shape denoting storage media (circle = ethanol, triangle = Bead solution) and extraction method). Abbreviations: PowerSoil/Bead solution (PB), PowerSoil/75% ethanol (PE), QIAamp/Bead solution (QB) and QIAamp/75% ethanol (QE).

We assessed whether the observed clustering of shared diversity by condition (Fig. 3), in addition to the sample’s overall diversity, were significantly influenced by storage time, storage media and extraction method by fitting linear mixed effects models (Supplementary Table 2). As a proxy for shared bacterial diversity between extracts, we used the PC2 values from Fig. 3. As a measure of overall alpha diversity, we used the Shannon Index. These models revealed that independent of the condition being tested, the shared (p = 0.47) and overall diversity (p = 0.24) did not significantly vary over 6 months of sample storage at −80 °C. The modelling did show that storage media and not extraction method had the biggest influence on the diversity of the oral microbiota recovered from dental plaque. For samples stored in Bead solution, extraction method did not influence the overall diversity recovered (p = 0.82, Fig. 4a), and these samples’ overall ecologies were similar as indicated by a high level of shared diversity (p = 0.89, Fig. 4b). Samples stored in Bead solution compared to ethanol, independent of whether a mechanical disruption extraction method was used, retrieved significantly higher diversity (PowerSoil p = 1.64E-07, QIAamp p = 0.0085, Fig. 4a) and had dissimilar overall ecologies as indicated by a low level of shared diversity (PowerSoil p = 0.0000237, QIAmp p = 0.0088, Fig. 4b). However, if samples were stored in ethanol, extraction of those samples with the QIAamp kit retrieved more diversity than samples extracted with the PowerSoil kit (p = 0.0008, Fig. 4a).

### Impact of storage media and DNA extraction method on species abundance

**Figure 5:**
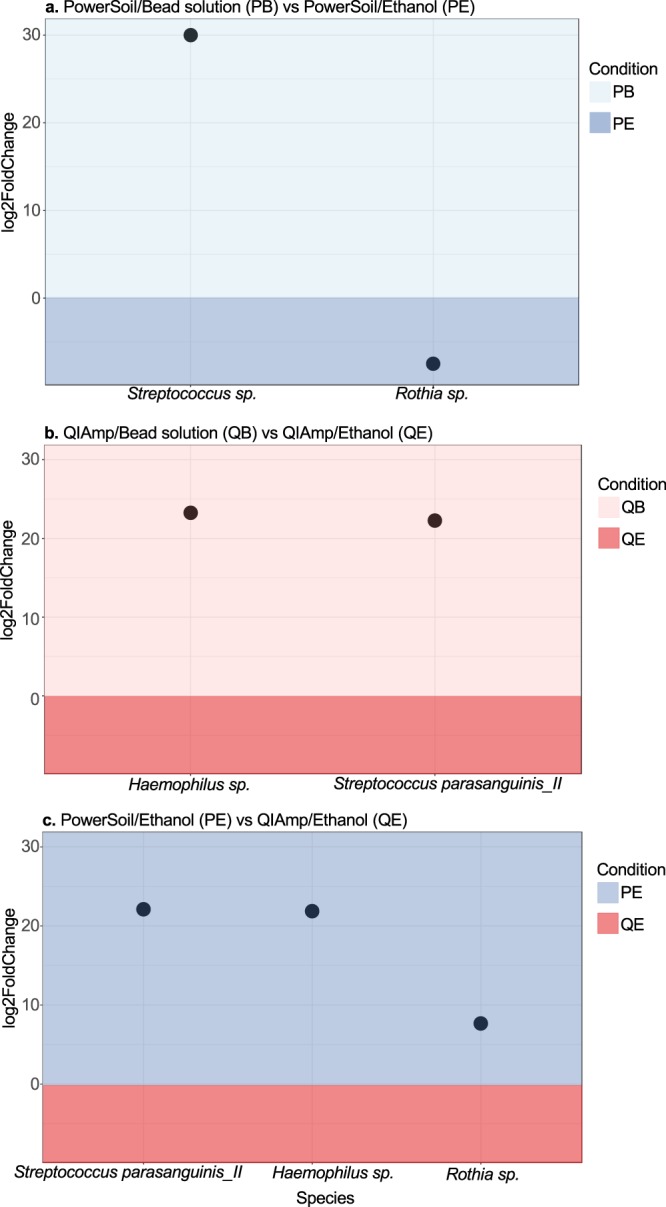
Differentially abundant species comparing storage medium and extraction method. DESeq2 was used to investigate if storage media and extraction kits influenced the abundance of ASVs in dental plaque samples, when adjusting for storage time (Phyloseq version 1.20.0). The figure shows species that were found to be significantly different (P < 0.001) between the two states (**a**) PowerSoil/Bead solution (PB) vs PowerSoil/Ethanol (PE), (**b**) QIAamp/Bead solution (QB) vs QIAamp/Ethanol (QE), and (**c**) PowerSoil/Ethanol (PE) vs QIAamp/Ethanol (QE). All p-values were adjusted for multiple comparisons using the Benjamini-Hochberg, False Discovery Rate procedure.

We also assessed whether storage media and extraction kit affected the stabilisation of specific bacteria. A differential abundance test, DESeq2^22,23^, was used to compare the abundance of ASVs present in dental plaque samples stored in Bead solution versus ethanol, and extracted with the PowerSoil versus the QIAamp kit, while controlling for length of time stored at −80 °C. Comparison of the four conditions revealed a similar profile of bacteria in samples from each condition, with only 4 of the 280 species being significantly differentially abundant between all states, (Supplementary Table 3). Similar to the diversity results, there was little difference between the Bead solution stored samples extracted with the PowerSoil to QIAamp kit. Only one species (*Streptococcus sp*., p = 5.26E-10) was significantly different, being more abundant in PowerSoil/Bead solution than QIAamp/Bead solution treated samples. In comparison, there were 4 differentially abundant ASVs between the Bead solution and ethanol-stored samples (Fig. 5). Of these four, three species were more abundant in Bead solution than ethanol-stored samples, independent of if the sample was extracted with PowerSoil (Fig. 5a) or QIAamp (Fig. 5b). These enriched species in Bead Solution included *Streptococcus sp*, *Haemophilus sp*. and *Streptococcus parasanguinis_II* (p < 0.001). For the ethanol-stored samples, those extracted with the PowerSoil compared to the QIAamp kit (Fig. 5c), recovered a higher abundance of *Streptococcus parasanguinis_II* (p = 6.09E-19), *Haemophilus sp*. (p = 8.11E-05) and *Rothia sp*. (p = 8.11E-05). The differential abundance test indicated that Bead solution stored and PowerSoil extracted samples recovered a higher abundance, particularly of streptococcus species, compared to ethanol-stored samples.

## Discussion

Biological sampling for big-scale, microbiome studies can be challenging because of large sample sizes, scale, and often geographic breadth. Thus, the availability of reliable preservation methods is essential to avoid changes in the microbial community being sampled, which is key for minimizing potential bias and enabling fair comparisons among bio-banked samples, especially for large initiatives, such as the Human Microbiome Project^21^. Numerous studies have compared different preservation methods for faecal samples^12,14,24^, yet the studies on dental plaque have centred around the evaluation of DNA extraction methods and 16S primers design^17,19,20,25^. In this study, we found that storage conditions, particularly storage media significantly influenced the overall diversity and ecologies of the oral microbiome samples, to a more substantial degree than the extraction method. Interestingly, a bead-beating, DNA extraction method did not appear to substantially improve the recovery of bacteria from dental plaque samples, as has previously been reported. Our results highlight the need to examine in combination the multiple steps involved in profiling oral microbiome samples, from storage to extraction, to assess where bias is potentially introduced.

Of the two storage media compared, Bead solution was found to be optimal to ethanol for dental plaque samples isolated with both DNA extraction kits tested, as it retrieved higher diversity and performed better at stabilising prevalent oral bacteria, such as *Streptococcus parasanguinis*. *Haemophilus sp*. and *Streptococcus sp* (Figs 4 and 5). Bead solution is the initial buffer used in the PowerSoil DNA Isolation kit, and is recommended by the Human Microbiome Project for storage of oral microbiome samples^26^, however, there has been no published studies evaluating the DNA preservation effect of this solution. Bead solution contains guanidine salts that are a protein denaturant and nucleic acid protector. While we found Bead solution to be a more effective storage medium compared to the more hazardous ethanol, ethanol has been far more widely used in molecular biology for microbial community stabilization and DNA preservation. This is because ethanol can inactivate enzymes such as DNAases and secondary metabolites, and it is globally accessible and low-cost relative to other stabilizers. Previous research using real time PCR found that storage in 70% ethanol at 4 °C protected the DNA integrity of oral bacteria^27^. We also found ethanol could stabilise a wide range of oral bacteria at a similar level of performance to Bead solution, and that a single species, *Rothia sp*. (p = 9.39E-07), was significantly more abundant in samples stored in ethanol than Bead solution (Fig. 5c). Potentially, a higher percentage of ethanol may have resulted in better preservation, as room temperature stored, faecal samples have been found to retain greater diversity when stored in 95% compared to 70% ethanol^14^. Our results indicate that for dental plaque, while Bead solution was a superior storage media, 75% ethanol was not substantially worse and is a suitable option, especially if cost is a limiting factor or *Rothia* species were of particular interest.

Of note, this is the first study to evaluate the DNA quality and integrity in dental plaque with different storage durations over a 6-month period using NGS. We found that compared with the freshly extracted plaque samples, the abundance, the shared and overall diversity of samples did not significantly vary over 6 months of storage at −80 °C (Fig. 3), indicating both ethanol and Bead solution preserved the DNA with high reliability. Our finding that the microbiome composition of dental plaque samples was stable at low temperatures over months is supported by shorter-time scale studies comparing oral microbiome samples at −80 °C for up to 2 weeks^28^ and longer-time scale studies comparing gut microbiome samples stored at −20 °C for up to 14 years^29^.

Interestingly, despite the focus on DNA extraction methods in the literature and the effect of chemical lysis and/or mechanical disruption via bead-beating, we found the isolation methods tested had little impact on the distribution of bacteria recovered from human dental plaque. For the Bead solution stored samples, both the bead-beating and chemical disruption method (PowerSoil) and the chemical only lysis method (QIAamp) retrieved nearly identical microbiome profiles from dental plaque, in terms of both diversity and abundance (Fig. 4). Our findings are consistent with previous studies showing a lack of impact of the addition of a bead-beating step on the microbiome composition from oral rinse^25^, saliva and plaque samples^20^. The addition of bead-beating has been found to improve the recovery of Firmicutes from oral rinse samples^30^, such as the oral pathogen *Streptococcus mutans*^19^. We also found the only significantly more abundant bacteria in the Bead solution samples extracted with bead-beating compared to without, was a Firmicutes species (*Streptococcus sp*.) (Fig. 5a). In comparison to our findings, studies based on mock communities have found that DNA extraction method exerted considerable influence on the observed bacterial diversity, retrieving higher diversity with an additional bead-beating step^7,17,31^. The inconsistency between our findings and others on the influence of bead-beating may arise from the difference between the non-biofilm, mock communities^7,17,31^ and human oral microbiota samples used in the study design^25^.

We also found that the storage media and extraction method may interact. For example, for samples stored in ethanol, extraction with the QIAamp kit recovered higher bacterial diversity than extraction with the PowerSoil kit (Fig. 4a). However, amongst this higher diversity there was a significant loss of abundance of key oral bacteria with QIAamp compared to PowerSoil kit, including *Streptococcus parasanguinis*. *Haemophilus sp*. and *Rothia sp* (Fig. 5c). This indicates that the ethanol may interact with buffers in the PowerSoil kit to negatively influence overall diversity, but that the addition of bead-beating still assists to recover important oral bacteria.

To ensure our findings were generalisable to a wider population and accurate, we assessed the influence of both biological and technical variation^32,33^. Biological variation was captured by sampling 20 individual’s dental plaque. The influence of inter-individual variation was strong, accounting for the majority of variation in shared phylogenetic diversity amongst extracts (68.1%, PC1) (Fig. 3), compared to the storage and extraction method (14.1%, PC2) (Fig. 4a). The strong influence of inter-individual variation on bacterial community composition has been previously found^34–36^. The finding of the influence of biological variation highlights the need to incorporate multiple individuals in microbiome methods testing studies, as opposed to one individual replicated multiple times^37^. For the purpose of assessing technical variation, all conditions for the second group of pooled samples were duplicated, with intra-condition variation playing a minor role.

Our study has limitations with regards to the sample size, use of a single region of 16S for sequencing and focus on bacteria as opposed to other microbial players. Our samples size is comparable (*n* = 20 individuals) to previous plaque and faecal microbiome studies that assessed between one^37^ and 15^14^ individuals. However, we did pool our samples into two groups to ensure adequate sample volume was available for the multiple comparison, an approach which has been used for prior oral and gut microbiota studies^12,13,15,20^. Secondly, our results are based on NGS with single target region of 16S (V4). However, Teng *et al*. has claimed that hypervariable regions have a relatively minor effect on the microbiome composition recovered and that the V4 region produced more reproducible results than other hypervariable regions (V1–V3)^19^. Finally, our analysis does not reveal whether storage media, storage duration and extraction kit would affect the preservation of other microbes, such as Fungi and Archaea.

In summary, our result showed that both Bead solution and ethanol can preserve bacteria from the oral microbiome in dental plaque with high reliability up to 6 months at −80 °C. Of the two storage media tested, Bead solution recapitulated the original picture of dental plaque, retrieving higher diversity and higher abundance of key oral bacteria, independent of using a DNA extraction with or without bead-beating. For ethanol stored samples, the QIAamp kit was superior at retrieving more overall diversity, but the PowerSoil kit retrieved a higher abundance of prevalent oral species. This study provides empirical evidence to researchers to assist with the selection of an appropriate storage media for dental plaque, especially for long-term storage, and the compatible extraction kit for DNA isolation, following which NGS can be performed to help broadening the knowledge on the role of oral microbiome in health and disease.

## Materials and Methods

### Ethics statement

Ethical approval for this study was granted by the Western Sydney Local Health District Human Research Ethics Committee (Refence NO. HREC/15/WMEAD/286). All methods were carried out in accordance with *Australian Code for the Responsible Conduct of Research*. Informed written consent was obtained from all participants.

### Subject selection and plaque sample collection

Subjects (age above 18, *n* = 20) were recruited and were systemically healthy, with no antibiotic use in the previous 6 months and were periodontally healthy. Informed written consent from each individual was obtained before the collection procedure. The participants were asked to refrain from smoking, eating and drinking for at least 30 minutes before the sample collection. A full mouth supragingival plaque sample was collected using a Catch-All^TM^ Sample collection swab (Epicentre) for each jaw. Each quadrant was rubbed on all tooth surfaces as many strokes as possible for 30 seconds. Immediately after swabbing, each swab was swirled in 1 × TE buffer for 30 seconds to ensure transfer of bacteria from swab to solution. The swab sponge was pressed against the tube wall multiple times. The samples were immediately transported on ice to the laboratory for further processing.

To create a homogenous sample with adequate volume to test the multiple conditions, the subject samples were split into two groups of 10. All dental plaque samples in TE buffer from a group were pooled (e.g. group 1 and group 2) into a 50 mL sterile Polypropylene Tube and vortexed until a consistent solution was achieved. The pooled plaque homogenates were divided separately into 200 uL aliquots for subsequent testing. All conditions were tested on both groups, with group 2’s conditions duplicated.

Overall four conditions were tested, including two storage media and two extraction methods, over four time points (Fig. 1). Plaque homogenate aliquots were centrifuged and pellets were either resuspended in 750 μL of 75% ethanol or 750 μL of PowerSoil® Bead Solution (Qiagen). The ethanol and Bead solution stored samples were then extracted with either the bead beating PowerSoil® DNA Isolation kit (Qiagen) or the chemical lysis QIAamp DNA Mini Kit (Qiagen). These four conditions were tested at the time of sample collection (time point 0), and after 1, 3, and 6 months storage at −80 °C.

### Genetic analysis

Genetic analysis of the dental plaque samples included DNA extraction, amplification of the 16S gene and sequencing of these amplicons on the Illumina MiSeq platform.

DNA extraction: At months 0, 1, 3, and 6, DNA extraction was performed using a 100-prep PowerSoil® DNA isolation kit and QIAamp DNA Mini Kit according to the manufacturer’s instructions respectively. For each method, an extraction blank (PCR-grade water) was used to ascertain potential kit and/or reagent contamination.

Amplification of the 16S region: PCR was used to amplify the V4 region (515–806) of the 16S rRNA gene. The forward primer sequence was GTGCCAGCMGCCGCGGTAA and the reverse primer was GGACTACHVGGGTWTCTAAT^38^. The PCR conditions included 0.625U of ThermoPol Taq (New England BioLabs) in a 25-μl volume using 10x ThermoPol Taq Buffer, 200 μM of each dNTP (Fermentas), 0.2 μM of each primer and 2 μl of DNA extract. The thermocycling conditions consisted of an initial enzyme activation step at 95 °C for 30 seconds, followed by 25 cycles of denaturation at 95 °C for 20 seconds, annealing at 54 °C for 15 seconds and elongation at 68 °C for 40 seconds, with a single final extension step at 68 °C for 5 minutes. Each set of PCRs included extraction and PCR blanks. Two microliters from each PCR products were visually examined by electrophoresis on a 2% agarose gel (w/v) containing GelRed DNA Gel Stain.

Illumina sequencing of the 16S amplicons: Illumina sequencing was used to examine the microbial profile of all sample DNA extracts. Amplicons were sequenced on the Illumina MiSeq platform with 250 base pair, paired-end read chemistry.

### Sequence analysis

Sequence analysis of the Illumina data, including quality filtering, taxonomic classification and phylogeny generation, was undertaken in QIIME version 2.2017.12^39^.

The Divisive Amplicon Denoising Algorithm (DADA2), a model-based approach, was used for correcting sequence errors and identifying biological variation at the level of single nucleotide differences^40^. The DADA2 model does not cluster sequences at a defined dissimilarity threshold, as was previously performed with Operational Taxonomic Unit clustering methods^39,41^. Sequences identified as not containing errors are referred to as ASV. This term is commonly used to describe sequences identified with high resolution denoising pipelines, such as DADA2^42^. Due to low sequence quality, the first 10 base pairs from the forward and reverse reads were removed prior to running DADA2.

Representative sequences from each ASV were taxonomically assigned using the Human Oral Microbiome Database (HOMD, version 14.51)^2^. A python version of the Ribosomal Database Project, Naïve Bayes classifier was used for taxonomic assignment^43^. Firstly, the classifier was trained on the same region of the 16S rRNA gene that was amplified from the samples (V4). Secondly, the trained classifier was tested on the representative sequences from the sample data. We reported taxonomic classification for sequences with a confidence score above 0.7. Representative sequences were aligned using MAFFT^44^, a de novo multiple alignment method, without a reference alignment. This alignment was used to build a phylogeny with fasttree^45^.

### Statistical analysis

Statistical analysis of the quality-filtered and classifier sequence data was undertaken in R (version 3.5.1).

Alpha diversity: Within-sample diversity was estimated per sample on the quality-filtered data, which had not been submitted to any further pre-processing, such as removal of singletons. The α diversity metric, Shannon Index, was calculated for all samples. To assess the impact of storage duration, storage media and DNA extraction method (together called ‘Condition) on α diversity, we used a linear mixed effects model, where the random effect was repeated sampling across time in R with the following formula lmer (Shannon Index ~ Storage Time + Condition + (1 | Sample)).

Pre-processing of sequences: Before undertaking further statistical analyses, very low abundant sequences were removed in line with current recommendations^46^. We removed ASVs that were singletons and had abundance below 0.01% of all the sequences.

Beta-diversity: Weighted unifrac analyses were performed on the rarefied sequence data (89, 927 sequences/sample) to compare the phylogenetic overlap between the different storage conditions and DNA extraction methods from two groups. The distances produced from the weighted unifrac were used for Principal Components Analysis to visualise the phylogenetic distance between ethanol and Bead solution stored samples, and those extracted with PowerSoil and QIAamp kits. We tested for the presence of a significant difference in phylogenetic distance between the four conditions using a linear mixed effects model in R with the following formula lmer (PC2 ~ Storage Time + Condition + (1 | Sample)). Given the clustering of samples by condition along the second principal components axis, the PC2 values were used as a proxy for testing condition effects on phylogenetic distance.

Differential ASV test: The DESeq2 package^22,23^ was used to test for the presence of differentially expressed ASVs between the four conditions in R using (version 1.20.0)^47^ on non-normalised and non-transformed data. The test is a negative binomial generalised linear model (GLM), Wald statistic was used to model the counts of ASVs per sample using a negative binomial distribution. The experimental design for the test was set to compare the combination of storage media and DNA extraction method (Condition), whilst taking into account variations between samples in storage duration (Storage_Time_months) and repeated sampling (Sample) using the following formula phyloseq_to_deseq2(Cond_biom_map_fil_100418, ~ Storage_Time_months + Sample + Condition). All ASVs that significantly (alpha = 0.01) differed in abundance between the conditions were reported. All reported p-values were adjusted for multiple comparisons using the Benjamini-Hochberg, False Discovery Rate procedure.

Normalisation of ASV count data: To account for variation in sequence depth between samples, we used Cumulative Sum Scaling (CSS) to produce normalised sequence data by library size with the MetagenomeSeq R package. All abundances of ASVs reported are from the CSS data.

## Supplementary information

Supplementary information

Supplementary Dataset 1

Supplementary Dataset 2

Supplementary Dataset 3

## Data Availability

The datasets generated during and/or analysed during the current study are available in the European Nucleotide Archive repository, accession number PRJEB34486.
