# Supplementary material for: Storage media and not extraction method has the biggest impact on recovery of bacteria from the oral microbiome
Source: Sci Rep. 2019 Oct 18;9:14968. doi: 10.1038/s41598-019-51448-7 (PMC6802381; doi:10.1038/s41598-019-51448-7)
Supplement: Supplementary file 1 — Supplementary information [file 41598_2019_51448_MOESM1_ESM.pdf]

Storage media and not extraction method has the biggest impact on recovery of bacteria from the oral microbiome

Xiaoyan Zhou, Shanika Nanayakkara, Jinlong Gao, Ky-anh Nguyen, Christina J Adler

Supplementary material

Supplementary figure 1. Sequencing depth per sample. The number of sequences in each dental plaque sample (ID) is coloured by condition (storage media and extraction method) and grouped by storage time (months).

Supplementary figure 2. Phylum abundance grouped by storage media, duration and extraction method. Phylum with a relative abundance above 2% are displayed, with the remainder of species grouped together (Phylum <2%) as boxplots, which show the mean and 95% confidence intervals for the data. The data have been normalised for sequence number via cumulative sum scaling (CSS). Abbreviations: PowerSoil/Bead solution (PB), PowerSoil/75% ethanol (PE), QIAamp/Bead solution (QB) and QIAamp /75% ethanol (QE).

Supplementary table 1. Abundance (%) of normalised (CSS) ASVs present in dental plaque samples.

Supplementary table 2. Linear mixed effects models for beta and alpha diversity. Beta diversity is calculated using PC2 values displayed in Figure 3. Alpha diversity values are calculated using the Shannon Index. Both alpha and beta diversity values were fitted to models using the same formula, lmer (a or beta diversity ~ Storage Time + Condition + (1 | Sample)).

Supplementary table 3. Differentially abundant species between storage media and extraction method. Deseq2 was used to run a single model, where the influence of storage media, extraction method and storage duration was assessed on ASV abundance. Four contrasts were pulled from the analysis to compare the four conditions: PowerSoil/Bead solution vs QIAamp/Bead solution, PowerSoil/Bead solution vs PowerSoil/Ethanol, QIAamp/Bead_solution vs QIAamp/Ethanol and PowerSoil/Ethanol vs QIAamp/Ethanol.

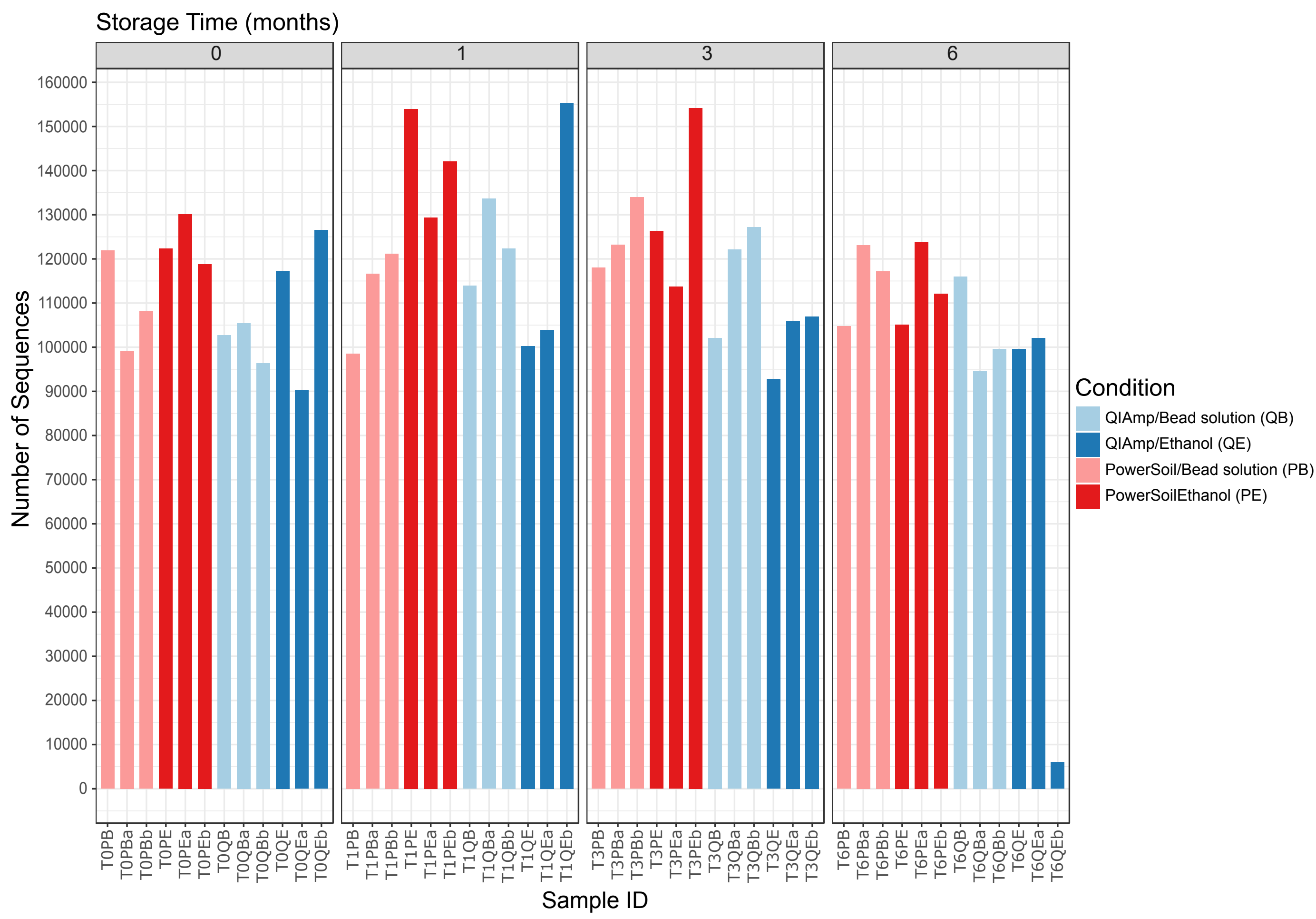
Storage Time (months)
0
1
3
6
Number of Sequences
Sample ID
Condition
QIAmp/Bead solution (QB)
QIAmp/Ethanol (QE)
PowerSoil/Bead solution (PB)
PowerSoilEthanol (PE)
T0PB
T0PBa
T0PBb
T0PE
T0PEa
T0PEb
T0QB
T0QBa
T0QBb
T0QE
T0QEa
T0QEb
T1PB
T1PBa
T1PBb
T1PE
T1PEa
T1PEb
T1QB
T1QBa
T1QBb
T1QE
T1QEa
T1QEb
T3PB
T3PBa
T3PBb
T3PE
T3PEa
T3PEb
T3QB
T3QBa
T3QBb
T3QE
T3QEa
T3QEb
T6PB
T6PBa
T6PBb
T6PE
T6PEa
T6PEb
T6QB
T6QBa
T6QBb
T6QE
T6QEa
T6QEb

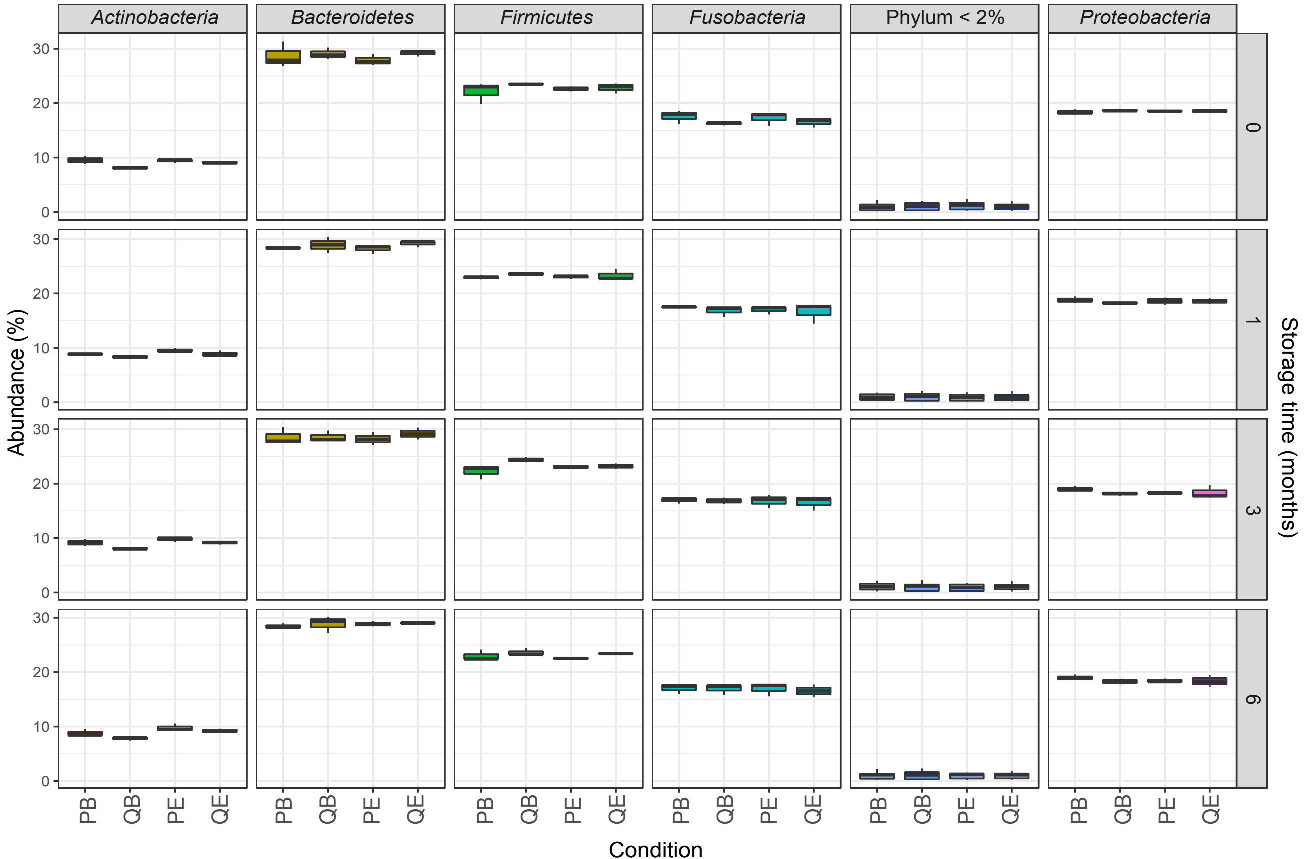
Actinobacteria
Bacteroidetes
Firmicutes
Fusobacteria
Phylum < 2%
Proteobacteria
0
1
3
6
Storage time (months)
Abundance (%)
30
20
10
0
PB
QB
PE
QE
Condition
